# Relationship between Hand Eczema Severity and Occupational Stress: A Cross-Sectional Study

**DOI:** 10.1155/2019/8301896

**Published:** 2019-10-08

**Authors:** Meriam Hafsia, Imene Kacem, Olfa El Maalel, Maher Maoua, Aicha Brahem, Haifa Aroui, Sana El Guedri, Houda Kalboussi, Souhail Chatti, Najib Mrizek

**Affiliations:** ^1^Department of Occupational Medicine, Sahloul Teaching Hospital, Sousse, Tunisia; ^2^University of Sousse, Faculty of Medicine of Sousse, Sousse, Tunisia; ^3^Department of Occupational Medicine, Farhat Hachad Teaching Hospital, Sousse, Tunisia; ^4^Department of Occupational Medicine, Ibn Jazzar Teaching Hospital, Kairouan, Tunisia

## Abstract

**Background:**

Stress has been recently implicated as a contributing factor of hand eczema (HE) severity. However, published data are both rare and contradictory justifying the need of further research. The purpose of this study was to evaluate the relation between stress and HE severity.

**Methods:**

This is a cross-sectional study enrolling all patients who have been attending the Dermato-allergology unit of Farhat Hached University Hospital of Sousse over a period of one year. The HE severity was assessed by the Osnabrück Hand Eczema Severity Index (OHSI). The stress level was assessed by the Perceived Stress Scale-10 (PSS-10) in its validated Arabic version.

**Results:**

During the study period, 109 participants meeting the inclusion criteria were identified. The mean age was 40 ± 9.9 years with a sex-ratio of 0.8. Severe eczema was found in 76 participants (69.7%). A high level of perceived stress was found in 18.3% of cases. A statistically significant association was noted between HE severity and the high level of perceived stress (*p*=0.039, OR = 4.46, 95% CI [0.96–20.59]) and the number of dependent children ≥3 (*p*=0.0039, OR = 1.92, 95% CI [0.51–7.22]). Leisure activity was found to be a protective factor against HE severity (*p*=0.031, OR = 0.27, 95% CI [0.09–0.80]).

**Conclusion:**

Although the link between the severity of eczema and atopy, wet work, and contact with irritants and allergens is well known, the relation remains questionable for other factors including stress.

## 1. Introduction

Hand eczema (HE) is a specific dermatosis because it touches the hand, main tool of work and communication, resulting in a variously felt discomfort [1].

Chronic and severe forms can have a serious impact on the patient's daily life, as it has individual and socioprofessional consequences [1]. Severe forms occur in 5 to 7% of cases and are likely to lead to major physical and psychological disabilities [2].

HE is a multifactorial pathology in which development and repetitive occurrence are closely linked to the environment. The clinical expression of the disease depends on living conditions, nature of the exposures, and genetic factors.

Factors involved in the onset of relapses and the transition to chronicity are numerous but not clearly identified. They can be endogenous and exogenous [3].

Many studies insist on the role of different factors exacerbating the HE. While a link between the severity of eczema and atopy, wet work, and contact with irritants and allergens is well known, the relation remains questionable for other factors including the stress, whether it is general or work-related [4, 5].

Studies on this subject are rare, and the results are contradictory, thus warranting further research so that firm conclusions can be drawn.

The objective of our study was to look for a possible relation between the severity of HE and occupational stress.

## 2. Methods

After obtaining the ethics committee approval and the oral patients consent, this cross-sectional and analytical study was performed in the dermato-allergology unit of Farhat Hached Teaching Hospital over one-year period (from 01/05/2017 to 31/05/2018).

Patients consulting for HE diagnosed by a dermatologist and exercising an occupational activity for at least one year were enrolled in this study.

Exclusion criteria were as follows: age under 18 years or above 65 years, patients with no lesions in the hands, and those with other diagnoses of hand dermatosis.

The diagnosis of ACD was clinically made and confirmed by a relevant patch test.

Data were collected using a medical questionnaire and a dermatological clinical examination. The medical questionnaire explored the sociodemographic characteristics (age, gender, educational level, and marital status), occupational data (sector of activity, occupational seniority, occupation, working in a humid environment, means of protection, nd occupational repercussions), lifestyle data (tobacco and alcohol consumption and extraprofessional activities), and medical information (family history of eczema, personal medical history, personal history of allergy, duration of eczema evolution, number of relapses per year, symptoms, and prescription of treatment).

The Perceived Stress Scale PSS-10 in its Arabic version was used to assess the stress level of our population. This scale is composed of 10 items divided into 2 groups. The first group has 6 negative items measuring “perception of stress,” while the second group has 4 positive items measuring “coping” or adaptation to stress. A high level of perceived stress is defined by a score more than 27 [6, 7].

Occupational stress was assessed by Siegrest's “effort-reward imbalance” questionnaire in its validated Arabic version [8]. This questionnaire is based on the imbalance between the efforts made and the expected rewards. It is a multidimensional tool composed of three scales: the Efforts Scale (5 items), the Rewards Scale (11 items), and the Overinvestment Scale (6 items). The calculation of the effort/rewards ratio is obtained by the following formula: ratio = 11/6 × extrinsic efforts/rewards. A ratio >1 defines employees exposed to an imbalance between efforts and rewards.

The dermatological clinical examination focused on the clinical appearance of hand eczema and the extent of lesions.

The HE severity was assessed by the Osnabrück Hand Eczema Severity Index (OHSI) [9]. This tool considers six morphological signs (erythema, scaling, papules, vesicles, infiltration, and fissures) and estimates their extent in the affected area by a four level scale (0 to 3). Severe hand eczema was defined as an OHSI score >7 [9].

Data were analyzed using SPSS.20.0. Student's “*t*” test was used to compare two independent series means. Comparison of frequencies was performed with Pearson's chi-squared test. Analyses of the relation between two quantitative variables (e.g., score and age) was done using Pearson's correlation coefficient.

Multivariate analyzes were performed using the logistic regression method to identify determinants of the severity of eczema; the dependent variable was severity of eczema (a binary variable with OHSI score > or ≤ 7), and the explanatory variables were all variables whose *p* value was less than or equal to 20% in univariate analysis. For all statistical tests, the significance level *p* was set at 0.05. The association was measured by the odds ratio (OR) that was presented with its 95% confidence interval.

## 3. Results

During the study period, 109 patients with HE were included. The average age was 40 ± 9.9 years. HE was more frequent in women (53.2 %).The majority of patients were married (75.2%), and 56% had at least two children.

Seventy patients (15.6%) were working in the healthcare sector, thirteen in the textile industry (12%), and eleven in construction field (10.1%). Average job seniority was 12.9 ± 9 years. Regarding the influence of HE on work, only 8 patients (7.5%) were transferred from their workstation. Personal history of atopy, revealed by asthma, allergic rhinitis, or allergic conjunctivitis, was found in 25 patients (23%) without any atopic eczema condition. Sociodemographic and occupational data are summarized in Table 1. Severe HE was noted in 76 participants (69.7%) according to OHSI score.

Mean score of PSS-10 was 20.1 ± 8.4 ranging between 9 and 37. The majority of the patients reported a moderate stress level (62.4%). Comparing the efforts made to the rewards at the professional level, 51 participants (46.8%) felt unbalanced.

The results of the evaluation of the different levels of perceived and occupational stress among the participants are summarized in Table 2.

A significant association was observed between the severity of HE and the number of dependent children. A number of dependent children ≥3 was associated with a significant increase in the risk of HE severity (*p*=0.039, OR = 1.92, 95% CI = [1.10–7.22]). The practice of a leisure activity was associated with a significant decrease in the risk of severity of HE (*p*=0.031, OR = 0.27, 95% CI = [0.09–0.80]). Neither sector of activity nor workstation was significantly associated with the severity of eczema (*p*=0.71 and *p*=0.15, respectively).

However, a statistically significant association between occupational seniority and the severity of eczema was noted with no increased risk (*p*=0.009, OR = 1; 95% CI [1–1.04]).

Associations between the severity of HE and the sociodemographic data, lifestyle habits, medical data, and occupational data are summarized in Table 3.

Regarding the relationship between the severity of eczema and the different levels of stress, a statistically significant association was found between the severity of eczema and the high level of perceived stress (*p*=0.039, OR = 4.46; 95% CI = [1.04 to 20.59]).

Also, a positive significant correlation was found between the Osnabrück score and the PSS score (*p*=0.002, *r* = 30%).

**Figure 1:**
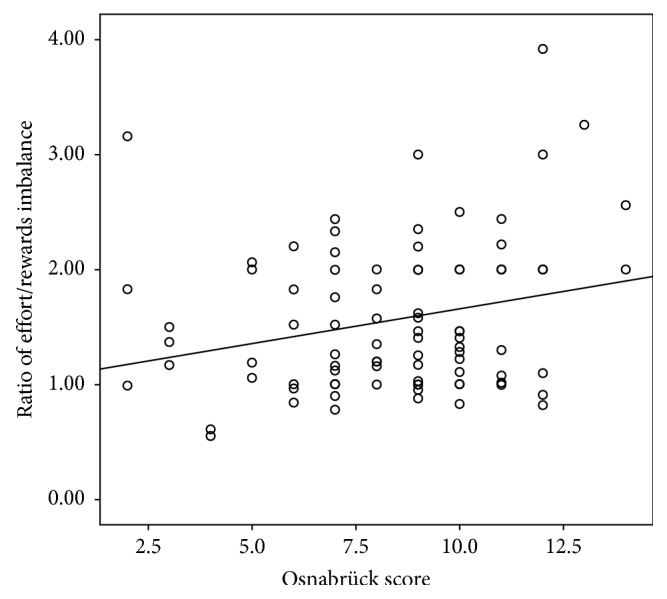
Correlation between the severity of HE and work stress.

In addition, a positive significant correlation was found between the Osnabrück score and the ratio of effort/rewards imbalance (*p*=0.009*p* = 0.009, *r* = 24%) (Figure 1).

Multiple binary logistic regression showed that the severity of HE did not seem to be associated with the studied variables.

## 4. Discussion

This cross-sectional epidemiological study carried out in the dermato-allergology unit of Farhat Hached Teaching Hospital of Sousse aims to assess the impact of occupational stress on HE severity.

The clinical evaluation of HE severity has an important role in daily clinical practice as well as in research. For various dermatological diseases such as atopic dermatitis and acne, many validated tools are available allowing an objective severity evaluation [10, 11]. However, for HE, there is no standardized scoring system. The clinical approach allows only a static evaluation of eczema severity and does not consider the fluctuations of its clinical presentation. Thus, a variety of grading systems are usually considered such as the Hand Eczema Severity index (HECSI) designed by Held et al. [12, 13]. This instrument is based on systematic measures of a combination of disease extent and clinical signs. Another approach was used by Coenraads et al. [14], who developed a simple five-point photographic classification system. In 2006, a third scoring system named “the Osnabrück Hand Eczema Severity Index” (OHSI) was published. Despite many similarities with HECSI, the OHSI seems to be simpler and easier allowing a wide use even by nondermatologists [9, 15].

In our study, the HE was evaluated by the OHSI score and was classified as severe in 69.7% of cases. Various results have been published depending on the evaluation methods used.

In the Hald et al.'s study [5], eczema was rated moderate to very severe in 60.3% of patients using the photographic and moderate guides using the “HECSI” score.

In a recent Danish study, 23.8% of respondents rated their current HE as moderate to very severe using the same photographic guide [16].

In most previous studies on risk factors of HE, the focus has been on atopy, skin irritation, and contact dermatitis. In this study, the relationship between some lifestyle factors and the severity of HE was sought.

The severity of HE was correlated with a high level of perceived stress and a number of dependent children ≥3. Leisure activity had been considered as a protective factor. In our study, we found a correlation between the Osnabrück score and effort/reward imbalance suggesting an influence of occupational stress on the severity of eczema. However, no significant association was found after multivariate analysis.

Factors associated with the severity of HE in the literature include advanced age, atopic dermatitis, and frequent flares in the last 12 months and at least 1 positive patch test [5].

Factors associated with a poor prognosis were frequent flares in the last 12 months and unskilled workers. In terms of morphology, fissures and desquamation were also identified as risk factors of poor prognosis [5].

In our study, only the number of dependent children (≥3) was associated with eczema severity. This could be explained by family constraints and the high mental and physical burden that could lead to a high level of stress, a delay in physician consultation, and a nonadherence to medication.

According to a German study, 48% of participants identified stress as a factor influencing their hand dermatosis. Respondents who reported high stress levels felt that their HE was more severe than those who had low levels of stress [17]. The relationship between stress and immune response in HE is not well studied. Our results suggested that the severity of HE could be influenced by a high level of perceived stress and occupational stress with an imbalance in the efforts provided compared to the rewards at the professional level in 46.8% of participants. However, the role of stress is difficult to assess because patient's ability of adaptation differs from one subject to another even for the same event [18].

Moreover, some published data still debate the above findings. In the study of Böhm et al. [19] involving 122 patients with work-related HE, eczema symptoms were not more severe in patients experiencing more stress and burnout despite their effects on patients ability of adaptation.

In fact, burnout syndrome is linked to chronic exposure to high levels of work stress leading to the exhaustion over time of physical abilities and cognitive and emotional resources that are important to resist negative consequences of stress on health [20].

There are few studies on hand dermatitis, specifically including occupational stress. Most of studies have been focalized in general stress such as family relationships or life events.

In a recent Danish study, enrolling 773 patients with a professional HE, no association was found between the severity of eczema and stress [4].

In. [17], 47.52% among all patients with hand dermatitis and contact dermatitis, Niemeier et al. reported that stress affects the course of their disease. According to published data, high patient stress response correlates with higher severity scores, more itching, and higher depression scores.

The correlation between stress and the onset and exacerbation of skin disorders that are commonly involved in HE is not well established [21, 22]. In fact, people who believe that their skin is sensitive to stress (perceived stress) report more serious symptoms, more severe depression, and more stressful life events [17]. HE has been shown to be more common among those reporting work-related stress [23], but the causal relationship between stress and symptoms has not been established. The psychological influences of stress on the severity and course of this disease have rarely been investigated [19].

As stress has a significant impact on the skin barrier [24], sebaceous secretion [25], cutaneous inflammation [26], and immunity [27], it logically participates in the occurrence of flares of inflammatory dermatoses. This has been demonstrated in psoriasis [28] or atopic dermatitis [29] but not yet in HE.

The psychological stress, frequently present in the professional world, worsens contact dermatitis, as it aggravates a large number of inflammatory diseases [30]. Mechanisms of the effect of stress have been studied in models of allergic contact dermatitis in mice subjected to nonpainful stress.

They showed that stress acts as an adjunct to inflammatory skin responses via norepinephrine released from the skin by the fibers of the sympathetic nervous system. The dendritic cells receiving this danger signal migrate more rapidly and in greater numbers to the ganglia, where they activate a larger number of effector T cells capable of inducing a larger and longer-lasting response [29]. Despite a marked exacerbation of several skin disorders by stress, the effect of stress on human skin has not been well studied [31].

According to a study of changes in the cutaneous barrier induced by stress, the authors concluded that stress could cause a delay in the recovery of cutaneous barrier function [31]. This study suggested that one of the mechanisms contributing to stress-induced exacerbation of certain chronic skin hyperplasias and inflammatory disorders was the stress-induced alteration of homeostasis of skin barrier permeability [31]. Disruption of the cutaneous barrier was associated with an increase in keratinocyte proliferation and in inflammation by cytokine production in the local tissue. Both of these effects could potentially trigger or aggravate inflammatory skin disorders such as eczema and atopic dermatitis and also aggravate contact dermatitis [31]. However, in some experimental studies, exogenous stress has been shown to have potent anti-inflammatory effects through increased endogenous glucocorticoids in mouse models with allergic contact dermatitis [32].

Although our study has the originality of treating the relationship between occupational stress and the severity of HE, an entity that has been rarely described, some limitations must be considered in interpreting the results. It is essentially the limited size of the sample and the relatively short period of the study.

In the same way, our study was interested only in patients who were consultants in the dermatology-allergology unit of Farhat Hached Teaching Hospital of Sousse, thus corresponding to a selected population. This selectivity is a bias towards the extrapolation of results to other populations, but in return, it facilitates the analysis of the data by reducing the heterogeneity of the studied population.

The use of self-questionnaires is a widely used approach in the field of medical research because of its speed and low cost. However, because of its declarative character, results can be influenced by participants' subjectivity. To remedy this deficiency, validated and standardized tools have been used to assess stress.

In our study, the subjective elements of HE were not considered in the assessment of eczema severity. It was performed via the Osnabrück Score which is a simple score that can be used by nondermatologists, particularly in epidemiological studies and in occupational health screening [15].

Finally, given the method used (cross-sectional study), the establishment of causal links between the variables studied could not be done. Furthermore, this study has targeted all the factors that have been incriminated in the literature in the severity of HE.

## 5. Conclusion

According to our study, HE severity seems to be associated with occupational stress. Thus, it is important to consider psychological factors in the follow-up and treatment of the patients. Psychological and educational interventions in addition to dermatological therapy should be an important basis for the management of HE. Similarly, at-risk patients must be identified and have access to training on coping strategies for occupational stress.

## Figures and Tables

**Table 1:** Sociodemographic and professional data of the participants.

Variables	Number of participants	Percentage

*Gender*		
Woman	58	53.2
Man	51	46.8

*Civil status*		
Single	24	22
Married	82	75.2
Divorced	3	2.8

*Level of education*		
Illiterate	7	6.4
Primary	34	31.2
Secondary	38	34.9
Academic	30	27.5

*Socioeconomic level*		
Low	2	1.8
Middle	87	79.8
High	20	18.3

*Number of children*		
0	34	15
1	14	6
2	28	4
3 and more	33	8

*Personal history of atopy*		
No atopy	84	77
Asthma	6	5.5
Allergic rhinitis	15	13.8
Allergic conjunctivitis	4	3.7

*Professional activity sector*		
Health	17	15.6
Administration	9	7.6
Construction and public works	11	10.1
Transport	9	8.5
Hairstyle and aesthetics	4	3.7
Chemical industry	15	13.7
Food industry	2	1.8
Wood, mechanical, and electrical industries	14	12.8
Hotel and catering trades	12	11.2
Textile	13	12.3
Others	3	2.7

*Occupation category*		
Administrative position	9	8.2
Employee	5	6.2
Labor	72	65
Technician	13	11.4
Doctor and paramedical staff	10	9.2

**Table 2:** Distribution of participants by level of stress.

Measuring tool	Number (*n*)	Percentage
*Score of PSS*		
<14	21	19.3
14–26	68	62.4
>27	20	18.3

*Effort/rewards imbalance (Siegrist)*		
Yes	51	46.8
No	58	53.2

PSS: Perceived Stress Scale.

**Table 3:** Association between severity HE and sociodemographic data, lifestyle habits, and medical and occupational data.

Variables	Severe HE		Nonsevere HE		*p* value	OR (95% CI)
	A ± SD		A ± SD			
Age (years)	39.8 ± 9.5		40.4 ± 10.7		*p* = 0.74	0.99 (0.95–1.03)
Duration of the complaint (years)	7.03 ± 6.22		7 ± 5.38		0.22	1.01 (0.95–1.08)
Number of relapses	4.57 ± 2.27		3.64 ± 2.74		0.06	1.19 (0.98–1.44)
Days off	11.5 ± 24.6		12.7 ± 23		0.97	0.99 (0.98–1.01)

	*N*	%	*N*	%		

*Gender*						
Woman	38	50	20	60.6	0.30	1.53 (0.67–3.53)
Man	38	50	13	39.4		

*Civil status*						
Single	15	19.7	9	27.3	0.25	1
Married	60	78.9	22	66.6		3.33 (0.26–42.21)
Divorced	1	1.4	2	6.1		5.45 (0.47–63.18)

*Children number*						
0	19	25	15	45.5	0.039	1
1	8	10.5	6	18.2		0.40 (0.14–1.15)
2	24	31.6	4	12.1		0.42 (0.11–1.60)
3 and more	25	32.9	8	24.2		1.92 (1.10–7.22)

*Sports activity*						
No	70	92.1	28	84.8	0.41	0.48 (0.13–1.70)
Yes	6	7.9	5	15.2		

*Leisure activity*						
No	69	90.8	24	72.7	0.031	0.27 (0.09–0.80)
Yes	7	9.2	9	27.3		

*Smoking status*						
No smoking	46	60.5	26	78.8	0.14	1
Current smoking	23	30.3	6	18.2		0.25 (0.02–2.16)
Weaned smoking	7	9.2	1	3		0.54 (0.05–5.35)

*Alcoholism*						
No	66	86.8	31	93.9	0.45	2.34 (0.48–11.36)
Yes	10	13.2	2	6.1		

*BMI*						
Normal	33	43.4	15	45.5	0.84	1.08 (0.47–2.47)
Overweight and obesity	43	56.6	18	54.5		

*Personal history of atopy*						
Yes	15	19.7	10	30.3	0.22	0.56 (0.22–1.43)
No	61	80.3	23	69.7		

*Clinical signs*						
Itching	67	88.2	31	93.9	0.65	1
Painful	5	6.6	1	3.1		0.54 (0.05–5.03)
Burning	4	5.3	1	3		1.25 (0.05–26.86)

*Treatment intake*						
Absent	14	18.4	4	12.2	0.62	1
Occasional	42	55.3	18	54.5		1.92 (0.50–7.29)
Daily	20	26.3	11	33.3		1.28 (0.51–3.22)

*Irritating products*						
Yes	44	57.9	15	18	0.23	1.65 (0.27–3.75)
No	32	42.1	45.5	54.5		

*Wet work*						
Yes	18	23.7	9	27.3	0.69	0.82 (0.32–2.09)
No	58	76.3	24	72.7		

*Frequent washing*						
Yes	31	40.8	17	51.5	0.30	0.64 (0. 28–1.47)
No	45	59.2	16	48.5		

*Hand protection*						
Yes	12	15.8	6	18.2	0.75	0.84 (0.82–2.48)
No	64	84.2	27	81.8		

*Occupational repercussions*						
Work resumption	69	90.8	29	87.9	0.89	1
Mutation	5	6.6	3	9.1		1.19 (0,10–13,64)
Other	2	2.6	1	3		0.83 (0.05–13.63)

A ± SD: average ± standard deviation.

## Data Availability

Readers can access the data underlying the findings of our study by contacting the corresponding author through mail.
